# Semidwarf Gene *d60* Affected by Ubiquitous Gamete Lethal Gene *gal* Produced Rare Double Dwarf with *d30* via Recombination Breaking Repulsion-Phase Linkage on Rice Chromosome 2

**DOI:** 10.3390/genes10110874

**Published:** 2019-10-31

**Authors:** Motonori Tomita, Jun Tanaka

**Affiliations:** 1 Research Institute of Green Science and Technology, Shizuoka University, 836 Ohya, Suruga-ku, Shizuoka 422-8529, Japan; 2 Faculty of Agriculture, Tottori University, 4-101 Koyama Minami, Tottori 680-8550, Japan; wildfowls@gmail.com

**Keywords:** rice, semidwarf gene, gamete lethal, non-Mendelian ratio, linkage, chromosome 2

## Abstract

The genotype of *gal* and *d60* were investigated in 33 rice varieties chosen from representative semidwarf and dwarf rice varieties. These were crossed with three tester lines, the *d60Gal* line (genotype *d60d60GalGal*), the *D60gal* line (Koshihikari, *D60D60galgal*), and the *D60Gal* line (*D60D60GalGal*). Each F_1_ plant was measured for culm length, and seed fertility. As a result, all F_1_ lines with the *d60Gal* line showed tallness and partial sterility, reduced by 25% in average from those with the *D60gal* line (Koshihikari) and the *D60Gal* line. These data indicated that the genotype of the 33 varieties is *D60D60galgal* and that the *d60* locus is not allelic to those of *sd1*, *d1*, *d2*, *d6*, *d18k*, *d29*, *d30*, *d35*, *d49*, *d50,* and *qCL1* involved in the 33 varieties. In addition, the *gal* gene is not complementarily activated with the semidwarf and dwarf genes described above, other than *d60*. The *Gal* gene will be ubiquitously distributed in rice. It is emphasized that *Gal* is a rare and valuable mutant gene essential to the transmission of *d60*. The double dwarf genotype of homozygous *d30d60* was rarely gained in the F_3_ of the *d30* line × *d60* line by breaking their repulsion *d60-D30* linkage on chromosome 2.

## 1. Introduction

The breeding program that has made the greatest contribution in the history of mankind is the ‘green revolution’ in which the production of grain was dramatically increased in the 1960s with the development of dwarf varieties of rice and wheat [1]. Dwarfing prevents plants from lodging at their full-ripe stage, which makes them lodging-resistant to wind and rain, and has enhanced their adaptability for heavy maturing, which has dramatically improved (up to double) rice yields, and so has contributed to the stabilization of yields all over the world. Surprisingly, semidwarf rice varieties developed independently using different native varieties or artificially induced mutant lines as mother plants, which are controlled by a single dwarf gene *sd1*. This is a defective C20-oxidase gene present in a late step in the gibberellin (GA) biosynthesis pathway [2], making options for dwarf breeding limited.

In order to find a novel dwarf gene to replace *sd1*, the first author conducted gene analyses focusing on Hokuriku 100, a mutant line with culms approximately 15 cm shorter than those of the Koshihikari variety. A novel dwarf gene, *d60*, was discovered, which gives rise to a good plant type with erect leaves by shortening culms by approximately 20%. Furthermore, *d60* complements the gametic lethal gene, *gal*, to cause gametic lethality [3]. For example, in the F_1_ hybrid (genotype *D60d60Galgal*) of Koshihikari (*D60D60galgal*) × Hokuriku 100 (*d60d60GalGal*), male and female gametes having both *gal* and *d60* become gametic lethal, and the pollen and seed fertility decrease to 75%. As a result, the F_2_ progeny show a unique mode of inheritance that is segregated into a ratio of 6 fertile long-culm (4*D60D60*:2*D60d60GalGal*):2 partially fertile long-culm (*D60d60Galgal* = F_1_ type):1 dwarf (*d60d60GalGal*). Moreover, the isogenic line that was introduced with both *d60* and *sd1* derived from Jukkoku [4,5] into Koshihikari by backcrossing [3], viz. the *d60sd1* line, and became the extreme-dwarf, indicating that *d60* is functionally independent from *sd1* and not related to the GA1 biosynthesis pathway [3]. Above all, *d60* is expected to diversify semidwarf breeding as a novel alternative of *sd1*. However, in the process of cross breeding, *d60* may cause gamete sterility if the counter parent has *gal*, and would result in an abnormal F_2_ segregation in an 8:1 ratio. Moreover, *d60* may affect the segregation of linked genes in the process of heredity. In this study we show: (1) the distribution of *gal* and *d60* were investigated in 33 representative semidwarf or dwarf varieties; and (2) double dwarfness of *d60* and linked *d30* was rarely gained from the F_3_ generation derived from the cross *d30* and *d60* line.

## 2. Materials and Methods

### 2.1. Test Crosses with Three Testers, d60Gal Line, D60gal Line, and D60Gal Line

In order to determine the genotype of *gal* and *d60* in 33 varieties chosen from representative semidwarf and dwarf rice, namely dwarf varieties with *d1* (Daikoku), *d2* (Ebisu), *d6* (Ebisumochi), *d18* Kotaketamanishiki), *d29* (Dwarf Kyushu 1), *d30* (Waisei shirasasa), *d35* (Tanginbozu), *d50* (Fukei 71), semidwarf varieties with *sd1* derived from Jukkoku (Jukkoku, Shiranui), semidwarf varieties with *sd1*derived from IR8 (Kinuhikari, Taichung 65 d47), semidwarf varieties with mutant *sd* induced by γ-ray-irradiation (Reimei, M101, HS90), semidwarf varieties with *qCL1* (Nipponbare), semidawarf varieties with unknown genes (Isehikari, Koganebare, Nihonmasari), uncharacterized dwarf mutants induced by *mPing* (IM96, IM181, IM265), artificial mutant strains of Koshihikari (Kanto 79 (with early maturing gene *e1*), Hokuriku 100 (d60)), and several long-culm varieties (Koshihsikri, Norin 1, Norin 22, Inochinoichi, Midoriyutaka, Ginbozu, Taichung 65, EG1) were used. These 33 varieties were crossed with the three tester lines, d60Gal line (*d60d60GalGal*), D60gal line (Koshihikari, *D60D60galgal*), and D60Gal line (*D60D60GalGal*). The d60Gal line was an isogenic Koshihiakri having *d60* and *Gal*, which was developed by seven times of continuous backcrossing with a recurrent parent Koshihikari and a non-recurrent parent of the *d60* homozygous segregant in the F_2_ of Koshihikari × Hokuriku100 [3]. The *D60Gal*-homozygous line was developed from F_4_ progenies fixed in the genotype *D60D60GalGal*, which derived from fertile and tall heterozygous F_2_ plants (*D60d60GalGal*), and segregated in the F_3_ according to the Mendelian segregation ratio of 1 (semidwarf (*d60d60GalGal*)):2 (1 semidwarf:3 tall *D60d60GalGal*):1 (tall *D60D60GalGal*) [6]. For each test cross combination between the 3 tester lines and 31 varieties, 10 F_2_ plants were cultivated at the Field Science Center. Seedlings were individually transplanted into a paddy field with densities 22.2 seedlings/m^2^ (one seedling per 30 × 15 cm). The paddy field was fertilized by 4.0 kg of basal fertilizer containing nitrogen, phosphorus, and potassium (weight ratio, nitrogen:phosphorus:potassium = 2.6:3.2:2.6) with 4.3 g/m^2^ nitrogen, 5.3 g/m^2^ phosphorus, and 4.3 g/m^2^ potassium dispersed evenly across the field.

### 2.2. Genotyping Using the Test Crossed F_1_ Lines 

Each F_1_ plant was measured for culm length and seed fertility. Three tester lines were isogenic lines in the genetic background of Koshishikari, which has a different single allele for *D60/d60* and *Gal/gal* loci, namely *d60Gal*, *D60gal,* and *D60Gal*. Taking into account the expectation that if the test subject has *gal*, F_1_ with the *d60Gal* line shows partial sterility, and both the F_1_ with the *D60Gal* line and the *D60gal* line shows fertility. On the other hand, if the test subject has a *d60 a*llele, the F_1_ with the *d60Gal* line shows dwarfness, the F_1_ with the *D60gal* line shows partial sterility, and the F_1_ with the *D60Gal* line shows fertility. Each of the F_1_ plants were scored with heading time and culm length in the field. The length between the ground surface and the panicle base of the main culm was measured as the culm length for all plants. The time when the tip of the panicle first emerged from the flag leaf sheath was recorded as the heading time for all plants. Three panicles were harvested from each F_1_ plant, and the number of filled and unfilled spikelets was counted for each panicle. The percent of seed fertility was calculated as the number of filled spikelets divided by the total number of spikelets multiplied by 100. The genotype was determined by seed fertility. Aceto-carmine squash mounts to stain the pollens of several F_1_ lines were conducted for Olympus BX40 microscopic examination.

### 2.3. Linkage Analysis for d60

**Figure 1:**
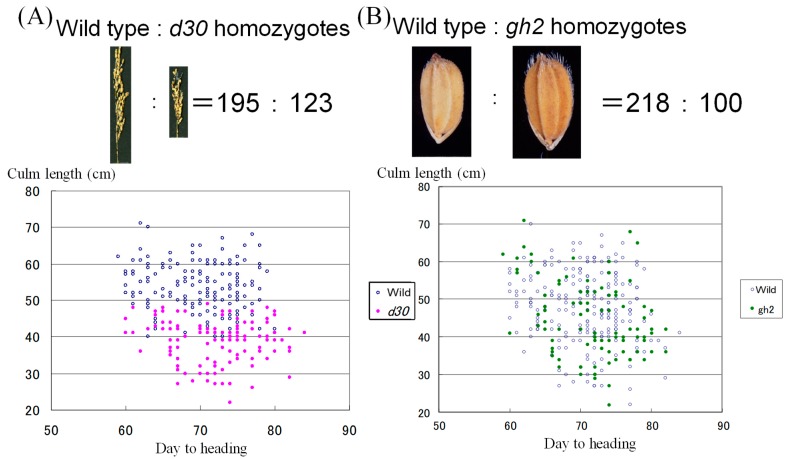
Excessive segregation of recessive homozygotes, according to the ratio of 5:4, considerably deviated from the Mendelian 3:1 ratio in the F_2_ between the recessive marker gene line FL212 and the *d60Gal* line (*d60d60GalGal*). (**A**) Genotyping for the *D30/d30* locus. *d30* homozygous plants showed characteristic dwarf phenotypes with short panicles and small grains. According to the dwarf trait, we visually discriminated the *d30* homozygote and wild type. As a result, the segregation ratio of wild type to *d30* homozygote at the *d30* locus was 195:123. In the correlation diagram with culm length and days to heading, red plots mean *d30* homozygous plants, whereas vacant plots mean wild type plants. (**B**) Genotyping for the *Gh2/gh2* locus. *gh2* homozygous plants showed characteristic gold-coloring of unhulled grain. *gh2* mutant is a lignin-deficient mutant, and *Gh2* encodes a cinnamyl-alcohol dehydrogenase [8] According to the gold color of the matured hull, we visually discriminated the *gh2* homozygote and wild type. As a result, the segregation ratio of wild type to *gh2* homozygote at the *gh2* locus was 218:100. In the diagram, green plots showed *gh2* homozygous plants, whereas vacant plots mean wild type plants. Above all, the recessive morphological gene *d30* and *gh2* on the chromosome 2, segregated in the characteristic ratio of 5 wild type:4 recessive homozygotes, suggesting their linkage with *D60* locus.

Firstly, 318 F_2_ plants of a marker gene line FL212 [7] that has *d30* and *gh2* on chromosome 2(*D60D60galgal*) and the *d60* line (*d60d60GalGal*) was used for segregation analysis for the marker genes and *d60*. The result showed that the segregation ratio of wild type to *d30* homozygote at the *d30* locus was 195:123, and wild type to *gh2* homozygote at the *gh2* locus was 218:100, and that both deviated significantly from 3:1 (Figure 1). When a recessive marker gene is fully linked to *D60*, the F_2_ segregation ratio of wild type to recessive marker gene homozygotes will be 5:4 (Supplementary File 1). This was closer to the expected ratio of 5:4 for the cases where *d30* is fully linked to *D60*. Then, F_3_ lines (50 individuals/lines) from 56 *gh2* homozygous F_2_ individuals from the cross between FL212 (*d30gh2)* and the Koshihikari d60 line were developed, and the genotype of the F_2_ was subsequently determined. 

## 3. Results

### 3.1. Universal Distribution of Gal and D60 Except for the d60 Donor Hokuriku100

**Figure 2:**
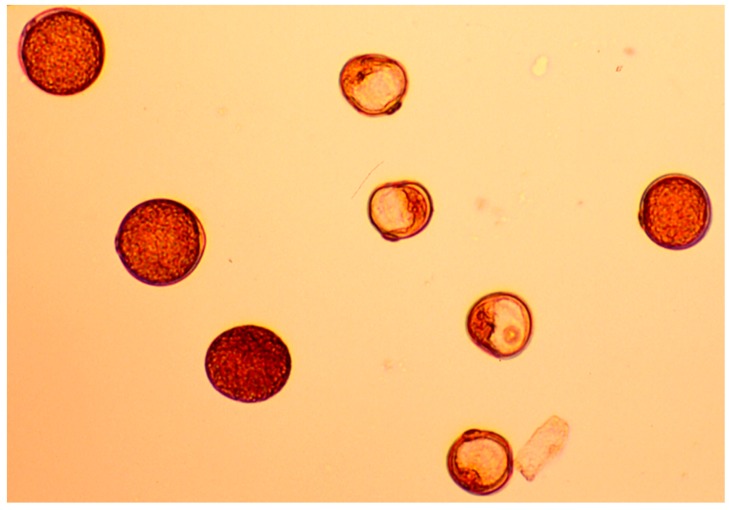
Partial pollen sterility in the F_1_ with the *d60Gal* line.

All F_1_ lines when crossed with the *d60Gal* line showed tallness and partial sterility, being reduced by an average of 25% from those with the *D60gal* line (Koshihikari) and the *D60Gal* line (Table 1, Figure 2). Regarding the dwarf varieties with *d1*, *d2*, *d6*, *d18*, *d29*, *d30*, *d35,* and *d50*, F_1_ lines with both of the *D60gal* line and the *D60Gal* line showed normal seed fertility over 90%, and there was statistically no significant difference between them. On the other hand, F_1_ lines with the *d60gal* line showed partial seed sterilities in the lower 70% level, which were reduced by approximately 25% from the F_1_s with the other two testers, namely the *D60gal* line and the *D60Gal* line, and the differences were statistically significant (5% level). Regarding to culm length, each of the F_1_ lines between the dwarf variety and the three testers showed almost the same normal length and there was statistically no significant differences between them. The above observations revealed that the all the dwarf varieties had *gal*, because the seed fertilities of F_1_s with the *d60Gal* lines were significantly reduced by 25% in accordance to the frequency of the genotype *d60gal* gametes. Furthermore, all the dwarf varieties did not have *d60*, because the seed fertilities of F_1_s with *D60gal* lines were at the normal 90% level, and culm length of the F_1_s with the three testers showed statistically the same level. Therefore, the genotype of the representative dwarf varieties for *D60Gal* loci were determined as *D60gal* homozygous.

The results of dwarf varieties were also true to the other varieties. Namely, regarding the semidwarf varieties with *sd1*derived from Jukkoku (Jukkoku, Shiranui), semidwarf varieties with *sd1*derived from *IR8* (Kinuhikari, Taichung 65 *d47*), semidwarf varieties with *sd1* mutant induced by γ-ray-irradiation (Reimei, *M101, HS90*), semidwarf varieties with *qCL1* (Nipponbare), semidawarf varieties with unknown genes (Isehikari, Koganebare, Nihonmasari), uncharacterized dwarf mutants induced by *mPing* [9] (IM96, IM181, IM265), artificial mutant strains of Koshihikari (Kanto 79 (with early maturing gene *e1*)), Hokuriku 100 (*d60*)], and several long-culm varieties (Koshihsikri, Norin 1, Norin 22, Inochinoichi, Midoriyutaka, Ginbozu, Taichung 65, EG1), the seed fertilities of the F1s with *d60Gal* lines were significantly reduced 25% from the lower 90% level of the F_1_ seed fertility with the other two testers, *D60gal* line, and *D60Gal* line. In addition, regarding to culm length, each of the F_1_ lines between these varieties and the three testers showed almost the same normal length, and there was statistically no significant difference between them. Therefore, the genotype of the semidwarf varieties with *sd1* for the *D60Gal* loci were determined as *D60gal* homozygous.

These data gave the following facts. The genotype of the 33 varieties is *D60D60galgal*, and the *d60* locus is not allelic to those of *sd1*, *d1*, *d2*, *d6*, *d18*, *d29*, *d30*, *d35*, *d49, d50, qCL1,* and unknown genes involved in the 33 varieties. In addition, the *gal* gene does not cause complementarily gamete lethality together with the semidwarf and dwarf genes other than the *d60* described above. Based on the above facts it is suggested that the *gal* gene will likely be distributed universally in rice. Therefore, it is emphasized that the *Gal* is rare, and is a valuable mutant gene essential to the transmission of *d60*.

### 3.2. Double Dwarfness with d30 and d60 Broken by Their Repulsion Linkage on Chromosome 2

**Figure 3:**
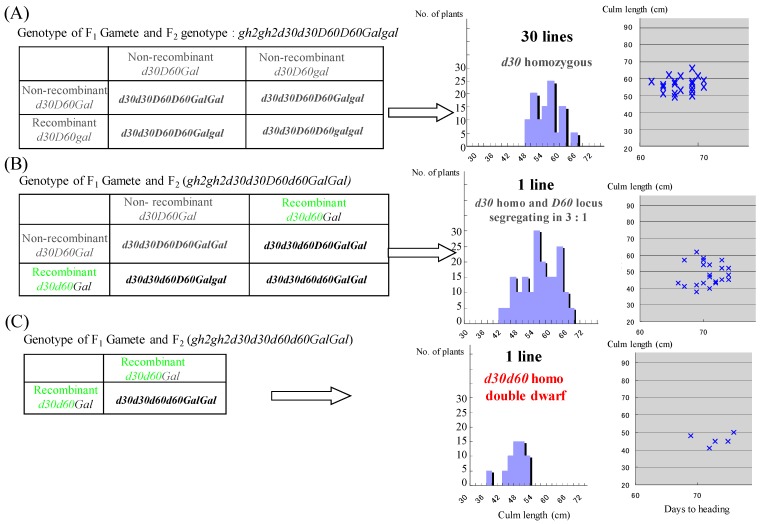
Genotyping of 32F_3_ lines derived from *d30gh2* homozygous F_2_ plants in the cross of *d30gh2* line *FL212* and Koshihikari *d60Gal* line. Each F_3_ line (50 individuals/line) was developed from 56 *gh2* homozygous F_2_ plants in the cross between the *gh2d30* line and Koshihikari *d60* line, and determined F_2_s’ genotypes. Thirty-two lines of *gh2d30* homozygous F_2_ plants were classified into three genotypes. (**A**) Thirty lines were homozygous of the non-recombinant gametes *d30*-*D60*, because the F_3_ progenies were fixed in the *d30* homozygous dwarf phenotype. (**B**) The single line has the recombinant gametes *d30*-*d60* and the non-recombinant gametes *d30-D60* in the heterozygous plant, because the *d30d60* double recessive phenotype appeared with approximately one fourth of the whole, namely, indicating a 3:1 segregation at the *d60* locus in the *d30* homozygous background. The green character represents the recombinant gametes. (**C**) The only single line was the *d30d60* double recessive dwarf, having the recombinant gametes *d30*-*d60* in the homozygous plant, because of its apparently shorter phenotype than the *d30* homozygous plant (Figure 4).

**Figure 4:**
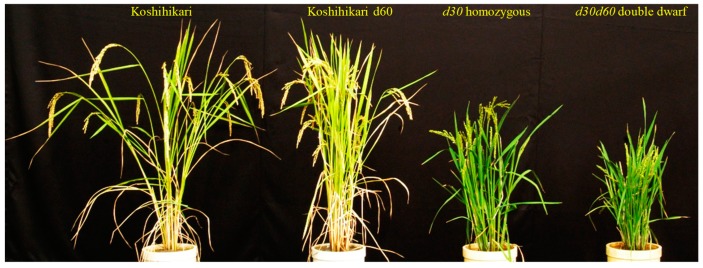
Phenotype of the *D30D60* homozygous wild type (Koshihikari), and homozygous plant for *d60D30, D60d30* and *d30d60* (left to right).

Each F_3_ line (50 individuals/line) was developed from 56 *gh2* homozygous F_2_ plants in the cross between *d30gh2* line and the Koshihikari *d60* line, and determined F_2_s’ genotypes (Table 2). First, 32 lines of *gh2d30* homozygous F_2_ plants were classified into three genotypes (Figure 3). Thirty lines were homozygous of non-recombinant gametes *d30*-*D60*, because the F_3_ progenies were fixed in the *d30* homozygous dwarf phenotype. The single line has the recombinant gametes *d30*-*d60* and the non-recombinant gametes *d30-D60* in the heterozygous plant, because *d30d60* double recessive phenotypes appeared with approximately one fourth of the whole, namely, indicating a 3:1 segregation at the *d60* locus in the *d30* homozygous background. Only one single line was a *d30d60* double recessive dwarf, having the recombinant gametes *d30*-*d60* in the homozygous plant, due to its apparently shorter phenotype than the *d30* homozygous plant (Figure 3 and Figure 4). 

**Figure 5:**
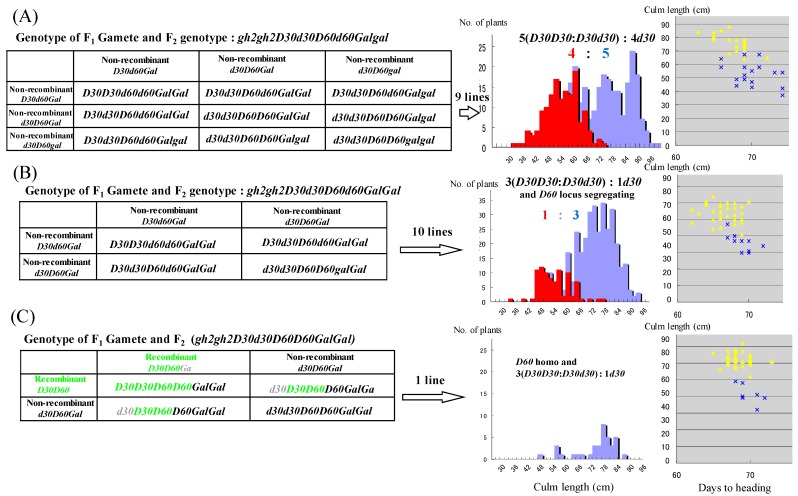
Genotyping of 20F_3_ lines derived from *D30d30gh2gh2* F_2_ plants in the cross of the *d30gh2* line FL212 and the Koshihikari *d60Gal* line. Twenty F_3_ lines having homozygous *gh2* and heterozygous *D30d30* were further classified into three genotypes. (**A**) Nine lines were heterozygous of the non-recombinant gametes *D60*-*d30*, *d60*-*D30,* and also for heterozygous *Galgal*, because these lines exhibited an excess segregation of the non-Mendelian 5:4 ratio at the *d30* locus together with partial sterility (214 (blue in histogram of culm length, yellow plot in the correlation diagram with culm length and days to heading):143 (red in histogram, blue plot in the diagram, χ^2^ = 2.786, 0.05 ≤ *p* ≤ 0.10)). (**B**) Ten lines were heterozygous for the non-recombinant gametes *D60*-*d30*, *d60*-*D30* and homozygous for *Gal*, because these lines segregated at the *d30* locus in the Mendelian 3:1 ratio (301:87, χ^2^ = 1.375, 0.10 ≤ *p* ≤ 0.90). (**C**) The single line has the recombinant gametes *D60*-*D30* and the non-recombinant gametes *D60-d30* in the heterozygous plant, because the line is segregated in a ratio of 3:1 at the *d30* locus. The green character represents the recombinant gametes.

Secondly, twenty lines having homozygous *gh2* and heterozygous *D30d30* were classified into three genotypes (Figure 5). Nine lines were heterozygous of the non-recombinant gametes *D60*-*d30*, *d60*-*D30* and also for heterozygous *Galgal*, because these lines exhibited an excess segregation of the non-Mendelian 5:4 ratio at the *d30* locus together with partial sterility, which is the same as in F_2_ (214:143, χ^2^ = 2.786, 0.05 ≤ *p* ≤ 0.10). On the other hand, 10 lines were heterozygous for the non-recombinant gametes *D60*-*d30*, *d60*-*D30,* and homozygous for *Gal*, because these lines segregated at the *d30* locus in the Mendelian 3:1 ratio (301:87, χ^2^ = 1.375, 0.10 ≤ *p* ≤ 0.90). The single line has the recombinant gametes *D60*-*D30* and the non-recombinant gametes *D60-d30* in heterozygous, because the line segregated in a ratio of 3:1 at the *d30* locus. Three lines were non–recombinant *d60-D30* homozygous and one line was heterozygous for recombinant gametes *d60*-*D30* and non-recombinant *D60-D30* gametes, and also for heterozygous *Galgal* (Table 2). These results indicated that *d60* is linked to *d30* with the recombination value calculated as 3.57% (= 4 recombinant gametes/112 total gametes × 100) on chromosome 2.

## 4. Discussion

The threat of strong typhoons due to global warming is increasing [10]. This is a serious problem in rice production, because strong winds cause stem lodging and consequent yield losses and deterioration in crop quality [11]. Extensive damage from the lodging of rice due to frequent typhoons has become a social problem in recent years, and developing new varieties of typhoon-resistant rice by introducing dwarf genes is an imperative task. Hence, there is a pressing need to develop new short-culm rice cultivars resistant to strong winds [12]. So far, *sd1* is the world’s only short-culm gene source in practical rice breeding. However, in the consideration of maintaining/expanding the genetic diversity of varieties, one should not rely only on *sd1*, which is a GA biosynthesis enzyme-defective gene, and should develop more new dwarf genes and promote their use in lodging-resistant breeding.

The excellent semidwarf quality of the rice mutant Hokuriku 100 is controlled by the single semidwarf gene *d60* [3,6]. It is desirable to generate lodging-resistant rice cultivars that carry a novel short-culm gene, *d60*, as an alternative to *sd1*. However, *d60* causes complementally gamete sterility, together with the gametic lethal gene *gal*. F_2_ progenies between *d60Gal* line (Hokuriku 100) and the original tall variety D60gal Line (Koshihikari) segregate distortedly into 1 semidwarf (*d60d60GalGal*):8 tall (2*D60d60Galgal*:2*D60d60GalGal*:4*D60D60*) ratio, because of the deterioration of the F_1_ male-and female-gametes having both *gal* and *d60* [6] (Supplementary File 1). In this study, the author developed F_1_ lines between 33 representative dwarf or semidwarf lines and three isogenic tester lines, the *d60Gal* line instead of Hokuriku 100 [13], the *D60Gal* line, and the *D60gal* line. Three tester lines were isogenic lines in the genetic background of Koshishikari, which were different in only a single allele for the *D60/d60* and *Gal/gal* loci, namely *d60Gal*, *D60gal,* and *D60Gal*. Therefore, when the test subject has *gal*, F_1_ with the *d60Gal* line shows partial sterility, and both the F_1_ with the *D60Gal* line and the *D60gal* line show fertility. On the other hand, when the test subject has *d60*, F_1_ with the *d60Gal* line shows as a semidwarf, F_1_ with the *D60gal* line shows partial sterility, and F_1_ with the *D60Gal* line shows fertility. As a result, all F_1_ lines with the *d60Gal* line showed tallness and partial sterility lower than a 70% level, whereas the F_1_ lines with the *D60gal* line (Koshihikari) and the *D60Gal* line showed a 90% level. In conclusion, the genotype of the 33 varieties was determined as *D60D60galgal*, and *d60* was different from *sd1*, *d1*, *d2*, *d6*, *d18*, *d29*, *d30*, *d35*, *d49, d50, qCL1,* and unknown genes involved in the 33 varieties. Moreover, there were no dwarf or semidwarf genes which were complementary with *gal,* except for *d60.*

The findings above suggest that the *gal* gene will likely be distributed universally in rice. Therefore, *d60* is a dwarf gene that could not have been obtained by chance without *Gal*’s simultaneous mutation. The *d60* gene could not have been transmitted without *Gal*. This means that the *Gal* gene is absolutely necessary to transmit *d60*, and *d60* is very unique in that it always makes a pair with *Gal* and segregates according to an 8:1 ratio. In other words, *d60* is a valuable gene because, without the *gal* to *Gal* mutation, *d60* would not exist in a normal environment.

The *d35* gene of Tanginbouzu, which became the best rice breed in Japan between 1955 and 1964, was kaurenoic acid oxidase- or 3-β hydroxylase-defective in the same GA biosynthesis pathway [14]. The Daikoku type dwarf gene *d1* in rice is defective in the α subunit of the heterotrimeric G protein, affecting GA signal transduction [15]. Both genes did not show complementary effects between *d60* and *gal*.

A progeny test was conducted in the F_3_ of the cross between the Koshihikari *d60* line and a line carrying a gene marker *d30* on chromosome 2, which when segregated in a ratio of wild-type to *d30* homozygote was 200:118, close to the theoretical segregation ratio of 5:4 at the *d30* locus when completely linked to the *D60* locus. This resulted in the genetic linkage between *d30* and *d60* loci on chromosome 2.

Here, we discuss the relationship between the complementary gamete sterility caused by *gal*, *d60*, and the previously reported hybrid gamete sterile genes in rice. Firstly, Oka [16] proposed that the duplicate *S* gene loci, which work as developmental factors in gametes, cause hybrid sterility when the F_1_ gametes receive both recessive *S* genes on each duplicate locus. For example, if parents A and B have genotypes *s1/s1 +2/+2* and *+1/+1s2/s2*, respectively, in which at least one + gene is necessary for normal development of the gamete, then 25% of their F_1_ hybrids will be sterile. This is because those gametes carrying the double recessive combination *s1s2* deteriorate due to deficiencies during gamete development. This hybrid sterility is similar to that caused by *gal* and *d60* in that two genes are responsible for both systems. However, *gal* and *d60* cause both sex sterilities, whereas Oka [17] suggests that the duplicate *S* gene model can only explain male gamete sterility.

On the other hand, Kitamura [18] explained female sterility in *indica/japonica* hybrids by the one locus sporo-gametophytic interaction hypothesis, that is, disharmony between one allele in the gamete and another in the surrounding sporophytic tissues. This model assumes parent genotypes of *S/S* and *S_a_/S_a_* creating the hybrid *S/S_a_*, in which the allele *S* present in the maternal tissue induces abortion of gametes carrying the opposite allele, *Sa*. Thus, 50% of *S/Sa* plants are sterile and produce gametes carrying the *S* allele only; selfed progenies are all fertile. Ikehashi et al. [19,20,21,22] showed that this one locus model was a more likely explanation for *indica/japonica* hybrid sterility than the two loci model [16]. The allelic interaction model [22] has been accepted as the genetic basis of hybrid sterility and the allelic interaction *S_5_* locus has been cloned [23].

In subsequent studies based on analyses of the fertility of a number of *indica* × *japonica* hybrids, over 30 female gametes sterility loci—including major genes—were identified and mapped [24,25,26,27,28,29,30,31,32,33], or male gametes sterility were identified [31]. So far, *indica/japonica* hybrid sterility loci were identified on chromosomes 4, 6, 7, 12, and 1 that lead to female gamete abortion through allelic interactions: *S_7_* [24], *S_8_* [25], *S_9_* and *S_15_* [27], and *S_16_* [26], etc. Among them, the *Sa* locus has been successfully cloned [34]. One-locus allelic interactions for male sterility were also recognized in hybrids between two cultivated rice species *Oryza sativa* and *Oryza glaberrima* Steud. [35,36,37], *O. sativa,* and *Oryza rufipogon* [38], and *O. sativa* and *Oryza. glumaepatula* [39], and a series of *S_1_* [37,40], *S_18_* [40], *S_20_* and *S_21_* [35,36], *S_22A_* and *S_22B_* [39], were identified. Above all, hybrid sterilities in rice can be explained by a single locus allelic interaction. Therefore, hybrid sterility caused by the two genes *d60* and *gal* is an extremely rare case in rice. Moreover, gamete breakdowns of both sexes, for *gal* and *d60*, are particularly rare and its ubiquities distribution is quite novel discovery.

## Figures and Tables

**Table 1:** Seed fertility (F) and culm length (C) of the F_1_ lines by crossing each variety and three testers, the *d60Gal* line, the *D60gal* line (Koshihikari) and the *D60Gal* line.

Variety		a		b		c					
	Dwarf Genotype	*d60Gal* Line		*D60gal* Line		*D60Gal* Line		*t*-Value between			
		*d60d60GalGal*		*D60D60galgal*		*D60D60GalGal*		a and c		b and c	
		F	C	F	C	F	C	F	C	F	C
Daikoku	*d1*	73.3	101.0	95.4	104.8	95.6	105.2	29.26 **	2.21	0.15	0.25
Ebisu	*d2*	72.4	94.2	95.1	98.3	94.8	98.2	27.56 **	2.43	0.58	0.43
Ebisumochi	*d6*	72.6	93.2	96.5	93.5	96.4	94.0	29.92 **	0.73	0.14	0.18
Kotaketamanishiki	*d18*	72.8	102.6	96.6	104.6	96.6	106.3	28.36 **	2.94	0.16	0.16
Dwarf Kyushu 1	*d29*	74.6	96.6	98.7	97.9	98.5	98.1	27.38 **	0.54	0.28	0.32
Waiseishirasasa	*d30*	73.9	82.3	95.9	83.7	95.6	84.1	28.24 **	1.34	0.32	0.28
Tanginbozu	*d35*	73.2	89.8	95.3	94.6	95.8	96.2	27.74 **	3.18 *	0.18	0.24
Fukei 71	*d50*	73.8	83.6	95.6	88.6	94.9	87.4	24.82 **	1.53	0.16	0.15
Jukkoku	*sd1*	72.8	84.7	96.4	88.5	96.6	87.8	26.66 **	1.33	0.22	0.18
Shiranui	*sd1*	72.4	83.2	95.6	86.3	95.2	86.5	29.46 **	1.48	0.52	0.56
M101	*sd1*	73.4	74.3	95.4	79.4	95.6	78.8	26.22 **	2.72	0.36	0.38
Taichung 65 d47	*sd1*	73.8	82.5	95.5	85.1	95.7	85.8	25.27 **	1.73	0.20	0.22
Kinuhikari	*sd1*	72.9	84.6	95.9	87.8	95.8	87.9	25.13 **	1.48	0.15	0.18
Reimei	*sd1*	72.7	75.8	95.2	78.3	95.7	78.4	25.46 **	2.18	0.49	0.56
HS90	*sd1*	72.9	73.6	95.3	76.2	95.8	76.3	25.32 **	0.67	0.18	0.20
Isehikari	unknown	73.2	86.1	95.8	88.3	94.7	89.0	26.77 **	1.89	0.77	0.68
Nipponbare	*qCL1*	73.3	75.4	94.7	78.9	94.5	79.6	27.82 **	3.17 *	0.23	0.17
Koganebare	unknown	74.2	80.0	95.4	88.7	94.7	85.8	27.55 **	20.9	0.33	0.36
Nihonmasari	unknown	73.4	78.6	95.6	84.6	95.1	84.7	26.82 **	3.38 *	0.24	0.28
IM96	unknown	73.8	93.1	96.2	93.4	95.8	92.8	25.67 **	0.18	0.17	0.34
IM181	unknown	72.5	82.3	95.6	84.4	95.5	84.2	25.46 **	1.23	0.24	0.26
1M265	unknown	73.8	81.9	96.0	82.7	95.7	82.9	28.37 **	1.16	0.18	0.19
Ginbozu		72.8	84.3	95.2	90.8	95.1	89.3	28.26 **	1.88	0.34	0.36
EG1		72.4	88.6	95.3	87.2	95.6	86.8	27.34 **	0.83	0.20	0.18
Taichung 65		73.1	97.1	96.3	97.8	96.2	97.2	25.68 **	1.08	0.24	0.28
Inochinoichi		72.8	98.3	96.7	102.3	96.5	102.8	29.35 **	2.28	0.78	0.68
Midoriyutaka		73.4	113.6	93.9	114.2	94.6	114.3	29.65 **	0.22	0.16	0.23
Yutakakoshihikari		72.5	75.3	96.5	78.2	96.4	77.9	25.86 **	1.34	0.27	0.22
Kanto 79	*e1*	73.5	76.7	96.9	77.0	96.8	76.8	27.25 **	0.35	0.26	0.28
Norin 1		72.2	96.7	96.3	96.7	97.3	95.8	30.64 **	0.98	0.26	0.28
Norin 22		73.7	75.3	95.6	77.8	95.4	78.0	28.39 **	1.23	0.25	0.24
Koshihikari	*D60*	73.6	72.7	96.2	76.6	96.6	76.4	25.64 **	1.91	0.18	0.15
Hokuriku 100	*d60*	95.6	62.8	73.4	72.8	96.5	72.6	0.15	11.26 **	26.78 **	0.14

* and **: Significant at 5% and 1% levels, respectively. All F_1_ lines when crossed with the *d60Gal* line showed tallness and partial sterility, being reduced by an average of 25% from those with the *D60gal* line (Koshihikari) and the *D60Gal* line. These data gave the following facts. The genotype of the 33 varieties is *D60D60galgal* and the *d60* locus is not allelic to those of *sd1*, *d1*, *d2*, *d6*, *d18*, *d29*, *d30*, *d35*, *d49, d50, qCL1,* and unknown genes involved in the 33 varieties. In addition, the *gal* gene does not cause complementarily gamete lethality together with the semidwarf and dwarf genes other than the *d60* described above.

**Table 2:** Genotyping of 56 F_3_ progenies derived from *gh2* homozygous F_2_ plants.

Genotype	No. of F_3_ Lines
*gh2gh2d30d30D60D60Galgal* non-recombinant + non-recombinant *gh2gh2d30d30D60D60GalGal* non-recombinant + non-recombinant	30
*gh2gh2d30d30d60D60Galgal* non-recombinat + recombinant	0
*gh2gh2d30d30d60D60GalGal* non-recombinant + recombinant	1
*gh2gh2d30d30d60d60GalGal* recombinant + recombinant	1
*gh2gh2D30d30D60d60Galgal* non-recombinant + non-recombinant	9
*gh2gh2D30d30D60d60GalGal* non-reconbinant + non-recombinant	10
*gh2gh2d30D30D60D60Galgal* non-recombinant + recombinant	0
*gh2gh2d30D30D60D60GalGal* non-recombinant + recombinant	1
*gh2gh2D30d30d60d60GalGal* non-recombinant + recombinant	0
*gh2gh2D30d30d60D60Galgal*recombinant + recombinant	0
*gh2gh2D30d30d60D60GalGal*recombinant + recombinant	0
*gh2gh2D30D30d60d60GalGal* non-recombinant + non-recombinant	3
*gh2gh2D30D30D60d60Galgal* non-recombinant + recombinant	1
*gh2gh2D30D30D60d60GalGal* non-recombinant + recombinant	0
*gh2gh2D30D30D60d60GalGal* non-recombinant+ recombinant	0
*gh2gh2D30D30D60D60GalGal*recombinant + recombinant *gh2gh2D30D30D60D60Galgal*recombinant + recombinant	0
	56

Recombinant value between *d30* and *d60* = 3.57%. According to the F_3_ genotyping as shown in Figure 4 and Figure 5, F_2_′s genotypes of 56 F_3_ lines were identified. The green characters represent the recombinant gametes. These results indicated that *d60* is linked to *d30* with the recombination value calculated as 3.57% (= 4 recombinant gametes/112 total gametes × 100) on chromosome 2.

## Supplementary Materials

The following are available online at https://www.mdpi.com/2073-4425/10/11/874/s1, Supplementary File 1: Identification of the chromosomal location of *d60* by distorted segregation of morphological marker genes, which linked with *D60*. If the recessive morphological gene is tightly linked with *D60*, segregation ratio of wild-type:recessive homozygotes is deviated to 5:4 from the Mendelian 3:1 ratio.

## References

[1] Khush G.S. (1999). Green revolution: Preparing for the 21st century. Genome.

[2] Sasaki A., Ashikari M., Ueguchi-Tanaka M., Itoh H., Nishimura A., Swapan D., Ishiyama K., Saito T., Kobayashi M., Khush G.S. (2002). Green revolution: A mutant gibberellin-synthesis gene in rice. Nature.

[3] Tomita M. (2012). Combining two semidwarfing genes d60 and sd1 for reduced height in ‘Minihikari’, a new rice germplasm in the ‘Koshihikari’ genetic background. Genet. Res. Cambridge.

[4] Tomita M. (2009). Introgression of Green Revolution sd1 gene into isogenic genome of rice super cultivar Koshihikari to create novel semidwarf cultivar ‘Hikarishinseiki’ (Koshihikari-sd1). Field Crops Res..

[5] Naito Y., Tomita M. (2013). Identification of an isogenic semidwarf rice cultivar carrying the Green Revolution sd1 gene using multiplex codominant ASP-PCR and SSR markers. Biochem. Genet..

[6] Tomita M., Tanisaka T. (2019). The gametic non-lethal gene *Gal* on chromosome 5 is indispensable for the transmission of co-induced semidwarfing gene *d60* in rice. Biology.

[7] Iwata N., Satoh H., Yoshimura A. (1989). Linkage map for Nishimura’s chromosome 8. Rice Genet. Newsl..

[8] Zhang K., Qian Q., Huang Z., Wang Y., Li M., Hong L., Zeng D., Gu M., Chu C., Cheng Z. (2006). Gold hull and internode2 encodes a primarily multifunctional cinnamyl-alcohol dehydrogenase in rice. Plant Physiol..

[9] Nakazaki T., Okumoto Y., Horibata A., Yamahira S., Teraishi M., Nishida H., Inoue H., Tanisaka T. (2018). Mobilization of a transposon in the rice genome. Nature.

[10] Knutson T., Camargo S.J., Chan J.C.L., Emanuel K., Ho C.H., Kossin J., Mohapatra M., Satoh M., Sugi M., Walsh K. (2019). Tropical Cyclones and Climate Change Assessment: Part I. Detection and Attribution. Bull. Am. Meteorol. Soc..

[11] Dahiya S., Kumar S., Chaudhary C. (2018). Lodging: Significance and preventive measures for increasing crop production. Int. J. Chem. Stud..

[12] Hirano K., Ordonio R.L., Matsuoka M. (2017). Engineering the lodging resistance mechanism of post-Green Revolution rice to meet future demands. Proc. Jpn. Acad. Ser. B.

[13] Tomita M. The gametic lethal gene *gal*: Activated only in the presence of the semidwarfing gene *d60* in rice. Proceedings of the 3rd Rice Genetics Symposium.

[14] Itoh H., Tatsumi T., Sakamoto T., Otomo K., Toyomasu T., Kitano H., Ashikari M., Ichihara S., Matsuoka M. (2004). A rice semi-dwarf gene, Tan-Ginbozu (D35), encodes the gibberellin biosynthesis enzyme, ent-kaurene oxidase. Plant Mol. Biol..

[15] Ueguchi-Tanaka M., Fujisawa Y., Kobayashi M., Ashikari M., Iwasaki Y., Kitano H., Matsuoka M. (2000). Rice dwarf mutant d1, which is defective in the α subunit of the heterotrimeric G protein, affects gibberellin signal transduction. Proc. Natl. Acad. Sci. USA.

[16] Oka H.I. (1953). The mechanism of sterility in the intervarietal hybrid (Phylogenetic differentiation of the cultivated rice plant. VI.). Jpn. J. Breed..

[17] Oka H.I. (1974). Analysis of genes controlling F1 sterility in rice by the use of isogenic lines. Genetics.

[18] Kitamura E. (1962). Genetic studies on sterility observed in hybrids between distantly related varieties of rice, *Oryza sativa* L.. Bull. Chugoku Nat. Agric. Exp. Stn. Ser. A.

[19] Ikehashi H., Araki H. (1984). Varietal screening of compatibility types revealed in F1 fertility of distant crosses in rice. Jpn. J. Breed..

[20] Ikehashi H. (1985). Theory and application for F1 fertility in remote cross of rice. Agric. Hortic..

[21] Ikehashi H., Araki H. (1988). Multiple alleles controlling F1 sterility in remote crosses of rice (*Oryza sativa*). Jpn. J. Breed..

[22] Ikehashi H., Bajaj Y.P.S. (1991). Genetics of hybrid sterility in wide hybridization in rice. Biotechnology in Agriculture and Forestry, Vol 14, Rice.

[23] Chen J., Ding J., Ouyang Y., Du H., Yang J., Cheng K., Zhao J., Qiu S., Zhang X., Yao J. (2008). A triallelic system of S5 is a major regulator of the reproductive barrier and compatibility of indica–japonica hybrids in rice. Proc. Natl. Acad. Sci. USA.

[24] Yanagihara S., Kato H., Ikehashi H. (1992). A new locus for multiple alleles causing hybrid sterility between an Aus variety and Javanica varieties in rice (*Oryza sativa* L.). Jpn. J. Breed..

[25] Wan J., Yanagihara S., Kato H., Ikehashi H. (1993). Multiple alleles at a new locus hybrid sterility between a Korean Indica variety and a Javanica variety in rice (*Oryza sativa* L.). Jpn. J. Breed..

[26] Wan J., Ikehashi H. (1995). Identification of a new locus S-16 causing hybrid sterility in native rice varieties (*Oryza sativa* L.) from Tai-hu Lake region and Yunnan Province, China. Breed. Sci..

[27] Wan J., Yamaguchi Y., Kato H., Ikehashi H. (1996). Two new loci for hybrid sterility in cultivated rice (*Oryza sativa* L.). Theor. Appl. Genet..

[28] Liu K.D., Wang J., Li H.B., Xu C.G., Liu A.M., Li X.H., Zhang Q. (1997). A genome-wide analysis of wide compatibility in rice and the precise location of the S5 locus in the molecular map. Theor. Appl. Genet..

[29] Wang J., Liu K.D., Xu C.G., Li X.H., Zhang Q.F. (1998). The high level of wide compatibility of variety ‘Dular’ has a complex genetic basis. Theor. Appl. Genet..

[30] Liu Y.S., Zhu L.H., Sun J.S., Chen Y. (2001). Mapping QTLs for defective female gametophyte development in an inter-subspecific cross in *Oryza sativa* L.. Theor. Appl. Genet..

[31] Song X., Qiu S.Q., Xu C.G., Li X.H., Zhang Q. (2005). Genetic dissection of embryo sac fertility, pollen fertility, and their contributions to spikelet fertility of intersubspecific hybrids in rice. Theor. Appl. Genet..

[32] Zhu S., Wang C., Zheng T., Zhao Z., Ikehashi H., Wan J. (2005). A new gene located on chromosome 2 causing hybrid sterility in a remote cross of rice. Plant Breed..

[33] Zhao Z., Wang C., Jiang L., Zhu S., Ikehashi H., Wan J. (2006). Identification of a new hybrid sterility gene in rice (*Oryza sativa* L.). Euphytica.

[34] Long Y., Zhao L., Niu B., Su J., Wu H., Chen Y., Zhang Q., Guo J., Zhuang C., Mei M. (2008). Hybrid male sterility in rice controlled by interaction between divergent alleles of two adjacent genes. Proc. Natl. Acad. Sci. USA.

[35] Sano Y., Chu Y.E., Oka H.I. (1979). Genetic studies of speciation in cultivated rice. 1. Genic analysis for the F1 sterility between *Oryza sativa* L. and Oryza glaberrima Steud. Jpn. J. Genet..

[36] Sano Y. (1983). A new gene controlling sterility in F1 hybrids of two cultivated rice species. J. Hered..

[37] Sano Y. (1990). The genetic nature of gamete eliminator in rice. Genetics.

[38] Sano Y. (1992). Genetic comparisons of chromosome 6 between wild and cultivated rice. Jpn. J. Breed..

[39] Sakata M., Yamagata Y., Doi K., Yoshimura A. (2014). Two linked genes on rice chromosome 2 for F1 pollen sterility in a hybrid between Oryza sativa and O. Glumaepatula Breed. Sci..

[40] Doi K., Yoshimura A., Iwata N. (1998). RFLP mapping and QTL analysis of heading date and pollen sterility using backcross populations between Oryza sativa L. and Oryza glaberrima Steud. Breed. Sci..

