# Supplementary material for: Semidwarf Gene d60 Affected by Ubiquitous Gamete Lethal Gene gal Produced Rare Double Dwarf with d30 via Recombination Breaking Repulsion-Phase Linkage on Rice Chromosome 2
Source: Genes (Basel). 2019 Oct 31;10(11):874. doi: 10.3390/genes10110874 (PMC6895840; doi:10.3390/genes10110874)
Supplement: Supplementary file 1 [file genes-10-00874-s001.pdf]

Morphological marker gene* line × Koshihiakri d60 line

*D60*D60galgal ↓ d60d60GalGal

F_1

*D60d60Galgal

↓

F_2

		F1 male gametes			
		*D60gal	*D60Gal	d60gal	d60Gal
F_1 female gametes	*D60gal	*D60*D60galgal	*D60*D60Galgal	No	*D60d60Galgal
	*D60Gal	*D60*D60Galgal	*D60*D60GalGal	No	*D60d60GalGal
	d60gal	No	No	No	No
	d60Gal	*D60d60Galgal	*D60d60GalGal	No	d60d60GalGal

Supplementary File 1. Identification of chromosomal location of *d60* by distorted segregation of morphological marker genes, which linked with D60.
If the recessive morphological gene* is tightly linked with *D60*, segregation ratio of wild type : recessive homozygotes* is deviated to 5:4 from the Mendelian 3:1 ratio.
* : recessive morphological gene tightly linked with *D60* allele
